# Enhanced Intracellular IR780 Delivery by Acidity-Triggered PEG-Detachable Hybrid Nanoparticles to Augment Photodynamic and Photothermal Combination Therapy for Melanoma Treatment

**DOI:** 10.1021/acsabm.5c00144

**Published:** 2025-04-12

**Authors:** Min-Chen Tsai, Lun-Yuan Hsiao, Yen-Hsuan Chang, Yu-Hsin Chen, Shang-Hsiu Hu, Chun-Yu Hung, Wen-Hsuan Chiang

**Affiliations:** † Department of Chemical Engineering, National Chung Hsing University, Taichung 402, Taiwan; ‡ Department of Biomedical Engineering and Environmental Sciences, National Tsing Hua University, Hsinchu 300, Taiwan; § Department of Orthopedic Surgery, Jen-Ai Hospital, Taichung 402, Taiwan; ∥ i-Center for Advanced Science and Technology (iCAST), National Chung Hsing University, Taichung 402, Taiwan

**Keywords:** PEG detachment, benzoic imine bond, IR780, photothermal and photodynamic therapy, melanoma treatment

## Abstract

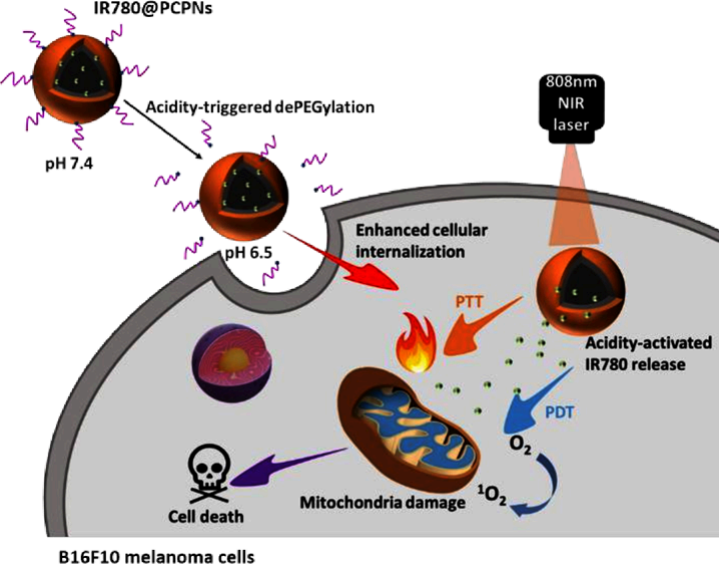

The PEGylation of drug-carrying nanoparticles has often been used to prolong blood circulation and improve drug deposition at tumor sites. Nevertheless, the PEG-rich hydrophilic surfaces retard the release of the payloads and internalization of therapeutic nanoparticles by cancer cells, thus lowering the anticancer efficacy. To boost the anticancer potency of the combined photodynamic therapy (PDT) and photothermal therapy (PTT) against melanoma by conquering the PEG dilemma, herein, the hybrid PEGylated chitosan-covered polydopamine (PDA) nanoparticles (PCPNs) with acidity-elicited PEG detachment ability were fabricated as carriers of IR780, a small-molecule photosensitizer used for PTT and PDT. The IR780@PCPNs displayed a uniform, solid-like spherical shape and sound colloidal stability. Under near-infrared (NIR) irradiation, the IR780@PCPNs showed prominent photothermal conversion efficiency (ca. 54.6%), robust photothermal stability, reduced IR780 photobleaching, sufficient singlet oxygen (^1^O_2_) production, and glutathione-depleting ability. Moreover, with the environmental pH being reduced from 7.4 to 5.0 at 37 °C, the decreased interactions between IR780 and PCPNs due to the increased protonation of phenolic hydroxyl residues within PDA and primary amine groups of chitosan accelerated the release of IR780 species from IR780@PCPNs. Importantly, the cellular uptake of IR780@PCPNs by B16F10 melanoma was remarkably promoted in a weakly acidic milieu upon PEG detachment driven by the disintegration of acid-labile benzoic imine. With NIR irradiation, the internalized IR780@PCPNs generated hyperthermia and ^1^O_2_ to damage mitochondria, thereby effectively inhibiting the proliferation of B16F10 cells. Collectively, our findings present a practical strategy for amplifying the anticancer efficacy of PTT combined with PDT using PEG-detachable IR780@PCPNs.

## 1 Introduction

Melanoma, an aggressive form of skin cancer, develops from skin melanocytes and is one of the most lethal diseases because of its high metastasis and resistance to chemotherapy.^1−3^ Melanoma is a rare tumor and less than 4% of skin cancer cases, but it accounts for 80% of skin cancer deaths.^4^ The treatment of patients diagnosed with early-stage melanoma using tumor excision and lymph node management is effective. Unfortunately, the five-year survival rate for advanced melanoma is only 16% due to the low sensitivity to traditional therapeutic modalities such as chemotherapy, radiotherapy, immunotherapy, and targeted therapy.^2,3,5^ Therefore, photodynamic therapy (PDT) and photothermal therapy (PTT) have emerged as new therapeutic tactics for melanoma treatment.^1−3,6−8^

PDT has received much attention in melanoma treatment due to its advantages, including noninvasiveness, high spatiotemporal control, low side effects, and convenience.^6−8^ Through the photochemical reaction of a photosensitizer excited with light of a specific wavelength to convert oxygen molecules in tumor tissues to toxic reactive oxygen species (ROS), PDT can induce tumor cell apoptosis and necrosis to inhibit tumor growth. However, the hypoxia and strong glutathione (GSH)-mediated antioxidant system of solid tumors largely limit the antitumor effect of PDT.^9−11^ More studies demonstrate that combining PDT with PTT is a viable approach to alleviate tumor hypoxia upon PTT-mediated local blood circulation increase and oxygenation.^10,12−16^ PTT relies on photosensitizers to transfer the absorbed light energy to generate heat and cause local hyperthermia, thus ablating target cancer cells.^16−18^ Recently, several commercial NIR dyes, including indocyanine green (ICG), IR780, and IR820, have been considerably used in PDT and PTT.^17−20^ Nevertheless, the poor water solubility, low photostability, insufficient photothermal effect, and inadequate cellular internalization of free photosensitizers have remarkably declined their clinical applications.^17−21^ To address these issues, various nanoparticles (e.g., liposomes, polymer-based micelles, nanoassemblies, silica-based nanoparticles, etc.) were fabricated as vehicles to realize the tumor-targeted delivery of photosensitizers.^17−20,22−26^ For example, Tang’s group fabricated IR780/lonidamine-carrying liposomes to enhance the anticancer efficacy of PDT/PTT combination therapy by inhibiting glycolysis to relieve tumor hypoxia.^22^ Furthermore, through coassembly of the amphiphilic diblock copolymer poly(lactic-*co*-glycolic acid)-*b*-poly(ethylene glycol) (PLGA-*b*-PEG) and electrostatic complexes comprising ICG molecules and branched poly(ethylenimine) (PEI), the ICG-loaded hybrid polymeric nanomicelles (PNMs) were developed by Jian and co-workers.^26^ The PNMs promoted the optical stability of ICG, significantly reduced ICG leakage, and enhanced intracellular ICG transport and photothermal cytotoxicity. Shin’s group developed PEGylated bovine serum albumin (BSA)-coated silver core/shell nanoparticles to deliver ICG for enhancing photothermal cancer therapy.^27^ In vitro studies showed that PEG-BSA-AgNP/ICG promoted ICG photostability. As reported by Li et al.,^28^ through the electrostatic, π–π stacking, and hydrophobic interactions, ICG and epirubicin (EPI) coself-assembled into small molecular nanoparticles, followed by surface coating with amphiphilic PEGylated lipid (1,2 distearoyl-*sn*-glycero-3-phosphoethanolamine-N-[methoxy(polyethylene glycol)-2000] (DSPE-PEG). The attained ICG-EPI nanoparticles exhibited high dual-drug loading, good physiological stability, superior photothermal response, pH-/photoresponsive drug release behavior, and promoted cellular internalization compared with free ICG or EPI.

The hydrophilic PEG-rich surfaces of most aforementioned nanoparticles not only enhanced their colloidal stability but also prolonged blood circulation via decreased capture by the reticuloendothelial system (RES), thus promoting the deposition of nanoparticles in tumor sites upon an enhanced permeability and retention (EPR) effect. In our previous works,^29,30^ to promote the colloidal stability of photothermal polydopamine (PDA) nanoparticles, the PEGylated chitosan conjugates were attained through the amidation of chitosan with PEG-COOH and coated on the surfaces of the as-synthesized PDA nanoparticles. The PEGylated chitosan-coated PDA nanoparticles were used as vehicles for Fe^3+^ and doxorubicin, respectively. Despite some advantages described above, the PEGylation of nanoparticles has been demonstrated to hinder the interaction of nanoparticles with target cancer cells, reducing cellular uptake and intracellular drug delivery.^31−35^ This would impact the anticancer efficacy. To conquer the PEG dilemma, in view of the weakly acidic microenvironment (pH_e_ 6.3–7.0) and hypoxia of tumor tissues, some functionalized PEG-based materials were decorated on the surfaces of the nanovehicles using the acid-labile bonds (e.g., hydrazone, orthoester, and benzoic imine bonds) or hypoxia-responsive bonds (e.g., azobenzene-4,4 ′-dicarboxylic acid),^31−35^ thus equipping these nanovehicles with tumor microenvironment-triggered dePEGylation capability. Despite significant advancements in developing nanoparticles with acidity- or hypoxia-triggered PEG detachment, most were utilized to deliver chemotherapy reagents or nucleic acid drugs.^31^ For all we know, only a few studies have reported using PEG-detachable nanoparticles to promote intracellular photosensitizer delivery for melanoma treatment,^33^ and these nanoparticles transport only a single PDT or PTT with limited anticancer potency.

**Scheme 1:**
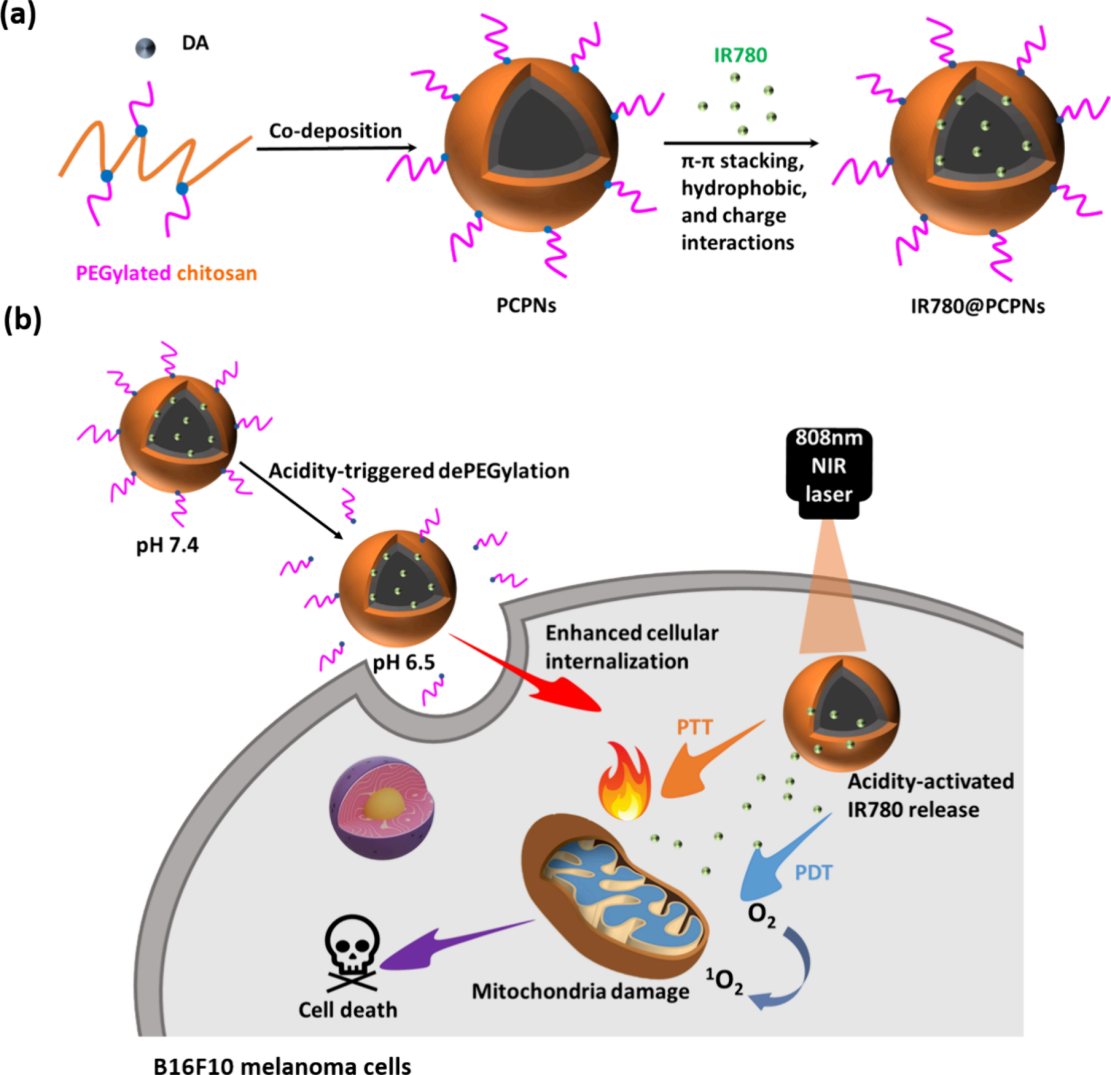
Illustrative Diagram of (a) Fabrication of IR780@PCPNs and (b) Their PTT/PDT-Based Anticancer Effect Enhanced by Acid-Activated PEG Detachment

Herein, we fabricated acidity-triggered PEG-detachable hybrid nanoparticles as carriers of IR780 to promote intracellular IR780 delivery for enhanced PDT/PTT-based melanoma treatment (Scheme 1). According to our previous work,^36^ as an essential component, the pH-responsive PEGylated chitosan adducts were prepared by conjugating methoxy PEG benzaldehyde (mPEG-CHO) with chitosan upon the acid-labile benzoic imine. Notably, different from the aforementioned PEGylated chitosan incapable of detaching PEG segments due to the lack of acidity-labile linkage,^29,30^ the PEGylated chitosan used in this study could realize the PEG detachment by the acid-cleavable benzoic imine. The hybrid PEGylated chitosan-coated PDA nanoparticles (PCPNs) were attained by simultaneous oxidative self-polymerization of dopamine molecules and the Michael addition of chitosan with PDA. Through the π-π stacking, hydrophobic, and charge interactions of IR780 molecules with PDA of PCPNs, the IR780-loaded PCPNs (IR780@PCPNs) with a satisfactory drug loading content (8.8 wt %) were obtained. The IR780@PCPNs exhibited a mean hydrodynamic diameter of ca. 166.9 nm and a solid-like spherical shape. The acid-induced disintegration of the benzoic imine allowed PEG segment detachment from the surfaces of IR780@PCPNs. Furthermore, the IR780 release from IR780@PCPNs was remarkably accelerated in response to a pH reduction from 7.4 to 5.0. Compared with free IR780 molecules, the IR780@PCPNs displayed superior photothermal conversion efficiency, colloidal stability, and photothermal stability. Also, IR780@PCPNs mediate depletion of GSH by the Michael addition between PDA and GSH and show ^1^O_2_-generating capability under NIR laser irradiation. Remarkably, under a culture environment of pH 6.5, imitating an acidic tumor microenvironment, the internalization of IR780@PCPNs by B16F10 melanoma cells was efficiently promoted via acidity-elicited dePEGylation. With NIR laser irradiation, the endocytosed IR780@PCPNs generated hyperthermia and sufficient ^1^O_2_ to damage mitochondria, thus leading to cell death (Scheme 1b). In conclusion, the acid-triggered PEG detachment of IR780@PCPNs can increase cellular uptake and boost PTT/PDT efficacy. This may provide a meaningful reference for future clinical melanoma treatment.

## 2 Materials and Methods

### 2.1 Materials

Dopamine·hydrochloride (DA) was acquired from Alfa Aesar (USA). Chitosan oligosaccharide (*M*_w_ 5.0 kDa, 81% degree of deacetylation) was obtained from Glentham Life Science Ltd. (UK). IR780 iodide dye (content 98%), 3-(4,5-dimethylthiazol-2-yl)-2,5-diphenyltetrazolium bromide (MTT), glutathione (GSH), 2’,7’-dichlorodihydrofluorescein diacetate (DCFH-DA), Dulbecco’s modified Eagle’s medium-high glucose (DMEM), Hanks’ balanced salt solution (HBSS), and D_2_O (99.9 atom % D) were purchased from Sigma-Aldrich (USA). 1,3-diphenylisobenzofuran (DPBF) and 5,5′-dithiobis (2-nitrobenzoic acid) (DTNB) were purchased from Fluorochem (UK). Fetal bovine serum (FBS) was obtained from Hyclone (USA). BODIPY 581/591 C11 was obtained from Thermo Fisher Scientific (USA). B16F10 cells (murine melanoma cells) and WS1 cells (human skin fibroblast cells) were acquired from the Food Industry Research and Development Institute (Hsinchu City, Taiwan).

### 2.2 Synthesis and Characterization of PEGylated Chitosan Adducts

The synthesis of mPEG-CHO and PEGylated chitosan and their characterization by FT-IR and ^1^H NMR were conducted based on the previously defined methodology.^36,37^ The PEGylated chitosan was prepared according to the synthetic route, as revealed in Figure S1.

### 2.3 Fabrication of IR780@PCPNs

DA molecules (2.5 mg) were dissolved in ethanol/deionized water cosolvent (0.8 mL). PEGylated chitosan adducts (6.0 mg) dissolved in tris buffer (0.2 mL) were added dropwise to the DA solution. After being stirred in the dark at 25 °C for 24 h, the resulting solution was dialyzed (Biomate MWCO 12,000∼14,000) against pH 8.0 phosphate buffer at 4 °C to obtain the purified PCPNs. Subsequently, IR780 in DMSO (2.0 mg/mL, 0.24 mL) was mixed with a PCPN-containing solution at pH 8.0 (5 mg/mL, 0.96 mL), followed by stirring in the dark at 25 °C for 24 h. The IR780@PCPN solution was dialyzed (Biomate MWCO 12,000∼14,000) against pH 8.0 phosphate buffer to eliminate unloaded IR780 molecules.

### 2.4 Characterization

The UV/vis spectra of DA monomers, PDA particles, PCPNs, free IR780 molecules, or IR780@PCPNs in pH 7.4 aqueous solutions were observed with a UV/vis spectrophotometer (U2900, Hitachi, Japan). The particle size and zeta potential of PCPNs and IR780@PCPNs in aqueous solutions were measured using a Litesizer 500 instrument (Anton Paar, USA). The structure analysis of PDA nanoparticles, PEGylated chitosan adducts, and PCPNs was conducted by X-ray photoelectron spectroscopy (XPS, PHI 5000 VersaProbe III X-ray photoelectron spectrometer. The morphology of PCPNs and IR780@PCPNs was attained by transmission electron microscopy (TEM) (JEM-1400 FLASH, JEOL, Japan) and scanning electron microscopy (SEM) (JEOL JSM-7800F Prime Schottky Field Emission SEM, Japan). The angle-dependent autocorrelation functions and mean-square radius of gyration (*R*_g_) of PCPNs and IR780@PCPNs were attained by the dynamic and static light scattering (DLS/SLS) measurements using a Brookhaven BI-200SM goniometer.

### 2.5 IR780 Loading Content and In Vitro IR780 Release

To quantify the IR780 amount of IR780@PCPNs, 50 μL of the IR780@PCPN solution before and after purification was diluted with deionized water to 1.0 mL. Afterward, the IR780 absorbance of the solutions at 780 nm was measured with a UV/vis spectrophotometer. The loading efficiency (LE) and loading content (LC) of IR780 were estimated as follows:
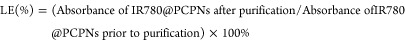

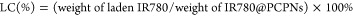


The in vitro IR780 dissolution test was performed as follows: 1.0 mL of aqueous solution of IR780@PCPNs was added to dialysis tubing (Biomate MWCO 12,000–14,000) and dialyzed against pH 7.4 and 6.5 PBS, and pH 5.0 acetate buffer (20 mL) at 37 °C, respectively. At the prescribed time intervals, the inner sample was taken out to analyze the maximum IR780 absorbance using the UV/vis spectrophotometer. Subsequently, the sample was returned to the dialysis tube. The cumulative IR780 release (%) was calculated based on the previously reported formula.^26^

### 2.6 Photothermal Performance and Stability

The temperatures and infrared thermographic maps of aqueous solutions with free IR780 molecules (22.2 μM), PCPNs (155 μg/mL), or IR780@PCPNs (170 μg/mL) were recorded during 808 nm laser irradiation (1.0 W/cm^2^) by using an infrared thermal imaging camera (Thermo Shot F20, NEC, Germany). Moreover, the temperature change of the above solutions during the cooling process was monitored. The photothermal conversion efficiency (η) can be estimated according to the formula previously reported elsewhere.^38,39^ To assess the photothermal stability, aqueous solutions of free IR780 molecules (22.2 μM), PCPNs (155 μg/mL), and IR780@PCPNs (170 μg/mL) were irradiated repeatedly with an 808 nm laser (1.0 W/cm^2^) for three cycles, each consisting of 5.0 min of laser exposure followed by 7.0 min of no laser exposure. After on/off laser irradiation each time, the absorption spectra of free IR780 and IR780@PCPN solutions were obtained with a UV/vis spectrophotometer. Also, the particle size of IR780@PCPNs before and after NIR laser irradiation was determined by a Litesizer 500.

### 2.7 ^1^O_2_ Generation and GSH Consumption

DPBF assay was used to assess the NIR-triggered ^1^O_2_ production of IR780 molecules and IR780@PCPNs. Free IR780 molecules and IR780@PCPNs (with an IR780 concentration of 66 μM) in a DPBF solution (0.89 mM) were exposed to laser irradiation (808 nm, 1.0 W/cm^2^) at different times, followed by the analysis of the DPBF absorbance using a UV/vis spectrophotometer. Moreover, the DPBF absorbance was normalized using the previously reported method.^17^

The GSH-depleting ability of PCPNs and IR780@PCPNs was evaluated by a DTNB assay. The PCPNs (400 μg/mL) and IR780@PCPNs (400 μg/mL) were each suspended in 110 mL of a 1 mM GSH solution at 37 °C for 2, 6, and 24 h, respectively, followed by centrifugation at 16,000 rpm for 10 min. A 0.9 mL aliquot of the supernatant was mixed with 0.1 mL of 1 mM DTNB and the absorbance at 412 nm was determined by a UV/vis spectrophotometer.

### 2.8 In Vitro Cellular Uptake

B16F10 cells (1 × 10^5^ cells/well) attached to 22 mm round glass coverslips in six-well plates were incubated in DMEM containing 10% FBS and 1% penicillin for 24 h. After the culture medium was discarded, the cells were incubated with the fresh culture medium containing free IR780 molecules at pH 7.4 or IR780@PCPNs at pH 7.4 and 6.5 (IR780 concentration: 2.5 μM) at 37 °C for 0.5 and 4 h. The fluorescence images at 405 and 780 nm excitation wavelengths for Hoechst and IR780 were attained with a confocal laser scanning microscope (CLSM) (Olympus, FluoView FV3000, Japan).

### 2.9 Intracellular ^1^O_2_ Generation

B16F10 cells (1 × 10^5^ cells/well) attached to round coverslips (22 mm) in 6-well plates were treated with PCPNs (17.4 μg/mL), free IR780 molecules, or IR780@PCPNs (IR780 concentration: 2.5 μM) at 37 °C and pH 7.4 or 6.5 for 4 h. Subsequently removing the medium, the cells were exposed to irradiation of 808 nm NIR laser (0.5 W/cm^2^) for 2.5 min or not. After being treated with DCFH-DA (5 μM) for 0.5 h, the cells were fixed using 4% formaldehyde, followed by observation of cellular images by fluorescence microscopy (ZEISS Axio Imager M2).

### 2.10 Mitochondrial Membrane Potential Examination

JC-1 staining was employed to assess the mitochondrial function. B16F10 cells (1 × 10^5^/well) in a 6-well plate were cocultured with PCPNs (17.4 μg/mL), free IR780 molecules at pH 7.4 or IR780@PCPNs (ICG concentration: 2.5 μM) at pH 7.4 and 6.5 for 4 h. After eliminating the medium, the cells were exposed to irradiation of an 808 nm NIR laser (0.5 W/cm^2^) for 2.5 min or not, followed by treatment with JC-1 (2 μg/mL) at 37 °C for 30 min. Afterward, the cells were fixed using 4% formaldehyde and observed by fluorescence microscopy. 485 and 535 nm excitation wavelengths were used to observe JC-1 monomer and JC-1 aggregate fluorescence. Moreover, the intracellular green fluorescence (JC-1 monomer) and red fluorescence (JC-1 aggregate) were quantified by ImageJ 1.53t, and the ratio of green and red fluorescence intensity was attained.

### 2.11 In Vitro Cytotoxicity

B16F10 cells (1.0 × 10^5^ cells/well) seeded in a 12-well plate were incubated with free IR780 molecules, PCPNs, or IR780@PCPNs of different concentrations at 37 °C and pH 7.4 or 6.5 for 4 h. After being separated with trypsin-EDTA and centrifuged (1500 rpm), the cells were collected and exposed to irradiation of an 808 nm laser (0.5 W/cm^2^) for 60 s or not. Then, the cells were redispersed in 0.65 mL fresh medium and incubated for 24 h. The cell viability was determined by MTT assay, as reported in our previous work.^17^ Furthermore, without NIR laser irradiation, the viability of healthy WS1 cells incubated with different formulations was assessed by a similar approach.

### 2.12 Statistics

Data were analyzed using GraphPad Prism software version 5.01 and presented as mean ± standard deviation. Two-way ANOVA analysis was conducted to obtain the differences of multiple groups. *p < 0.05, **p < 0.01, ***p < 0.001 were defined as significant differences.

## 3 Results and Discussion

### 3.1 Synthesis and Characterization of PEGylated Chitosan Adducts

**Figure 1:**
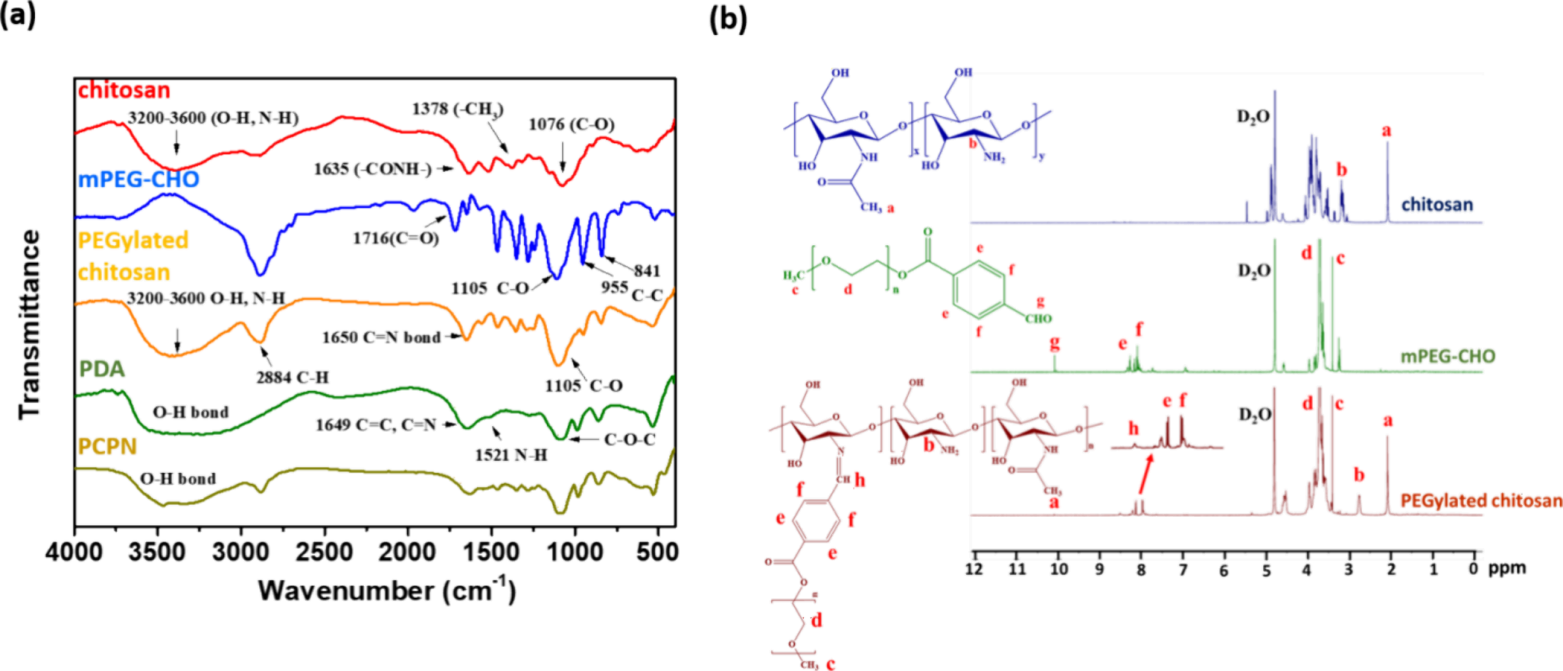
(a) FT-IR spectra of chitosan, mPEG-CHO, PEGylated chitosan, PDA, and PCPNs. (b) ^1^H NMR spectra of chitosan, mPEG-CHO, and PEGylated chitosan in D_2_O.

The PEGylated chitosan adducts were synthesized through a Schiff base reaction between mPEG-CHO and chitosan (Figure S1). As revealed in the FT-IR spectrum of PEGylated chitosan (Figure 1a), the feature bands of C–O, C–C stretching vibration from mPEG segments at 1105 and 955/841 cm^–1^ and C=N stretching vibration from imine linkages at 1650 cm^–1^, respectively, and the disappearance of the characteristic band of C=O stretching vibration at 1716 cm^–1^ from benzaldehyde groups of mPEG-CHO segments were observed. Also, the absorption band of the N–H/O-H and C–H from chitosan and mPEG at 3200∼3600 and 2884 cm^–1^ was attained, respectively. Note that the full vanishment of the aldehyde proton signals at δ 10.1 ppm and the appearance of the ethylene proton signals of mPEG at δ 3.7 ppm and imine proton at δ 8.5 ppm and glucosamine proton of chitosan at δ 2.8 ppm were obtained in the ^1^H NMR spectrum of PEGylated chitosan adducts (Figure 1b). These findings confirm that mPEG-CHO was efficiently conjugated with chitosan by forming benzoic imine bonds. Based on the integral ratio of the methoxy protons (δ 3.4 ppm) of mPEG-CHO and the H2 protons (δ 2.8 ppm) from glucosamine groups of chitosan, the number of mPEG chains for the one hundred glucosamine residues was calculated to be approximately 9.4.

### 3.2 Fabrication and Characterization of IR780@PCPNs

**Figure 2:**
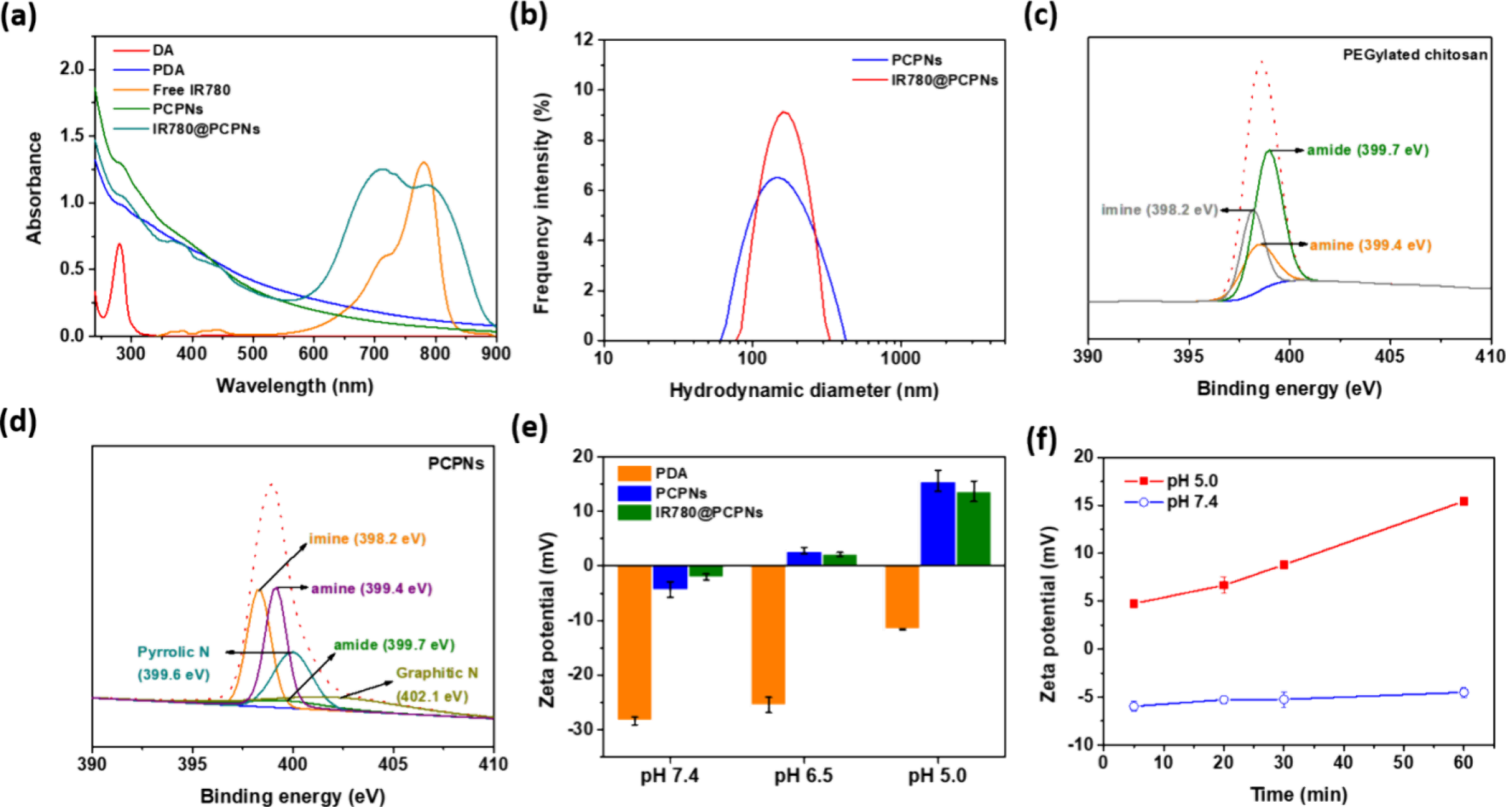
(a) UV/vis absorption spectra of DA monomers, PDA particles, free IR780 molecules, PCPNs, and IR780@PCPNs in pH 7.4 aqueous solution. (b) Particle size distribution profiles of PCPNs and IR780@PCPNs dispersed in pH 7.4 PBS. N 1s XPS spectra of (c) PEGylated chitosan adducts and (d) PCPNs. (e) Zeta potential values of PDA particles, PCPNs, and IR780@PCPNs at pH 7.4, 6.5, and 5.0. (f) Zeta potential values of IR780@PCPNs in aqueous solutions at pH 7.4 and 5.0 at different time intervals.

To attain PCPNs as IR780 vehicles, the DA molecules were dissolved in a PEGylated chitosan-containing aqueous solution and self-polymerized via oxidation. As revealed in Figure 2a, the DA molecules revealed only a feature absorption peak at 281 nm, while the PCPNs showed appreciably increased absorption from 300 to 900 nm, similar to bare PDA. Also, the PCPNs dispersed in pH 7.4 PBS exhibited a monomodel size distribution (PDI: 0.21) and an average hydrodynamic diameter of ca. 155.8 nm (Table 1 and Figure 2b). By contrast, in the lack of PEGylated chitosan adducts, the oxidative self-polymerization of DA molecules tended to form large PDA particles, thus forming significant precipitates (Figure S2). These results suggest that the PCPNs could be successfully prepared by one-pot codeposition of DA molecules and PEGylated chitosan adducts and further stabilized by hydrophilic PEGylated chitosan adducts. Compared to the XPS spectra of PEGylated chitosan and PDA (Figures 2c and S3), the XPS spectrum of PCPNs has the feature peaks of pyrrolic N (399.6 eV) and graphitic N (402.1 eV) from PDA and of amine (399.4 eV), amide (399.7 eV) and imine (398.2 eV) from PEGylated chitosan adducts (Figure 2d). Based on these findings, through the Michael addition between primary amine groups of chitosan with 5,6-dihydroxyindole units of PDA, the PEGylated chitosan adducts were effectively conjugated with PDA nanoparticles (Scheme 1a). Based on the TGA profiles (Figure S4), the PCPNs comprise approximately 74.1 wt % PEGylated chitosan and 25.9 wt % PDA. Moreover, the zeta potential of PCPNs at pH 7.4, obtained to be around −5 mV, is distinct from that of the bare PDA nanoparticles (ca. −28.5 mV) (Figure 2e). This suggests that the hydrophilic PEGylated chitosan significantly shields the negative charges from the phenolic hydroxyl groups of PDA.

**Table 1:** DLS Data and IR780 Loading Capacities of PCPNs and IR780@PCPNs

sample	*D*_h_ (nm)	PDI	LE (%)	LC (wt %)
PCPNs	155.8 ± 2.5	0.21 ± 0.02		
IR780@PCPNs	166.9 ± 5.6	0.20 ± 0.04	96.7 ± 2.4	8.8 ± 0.2

**Figure 3:**
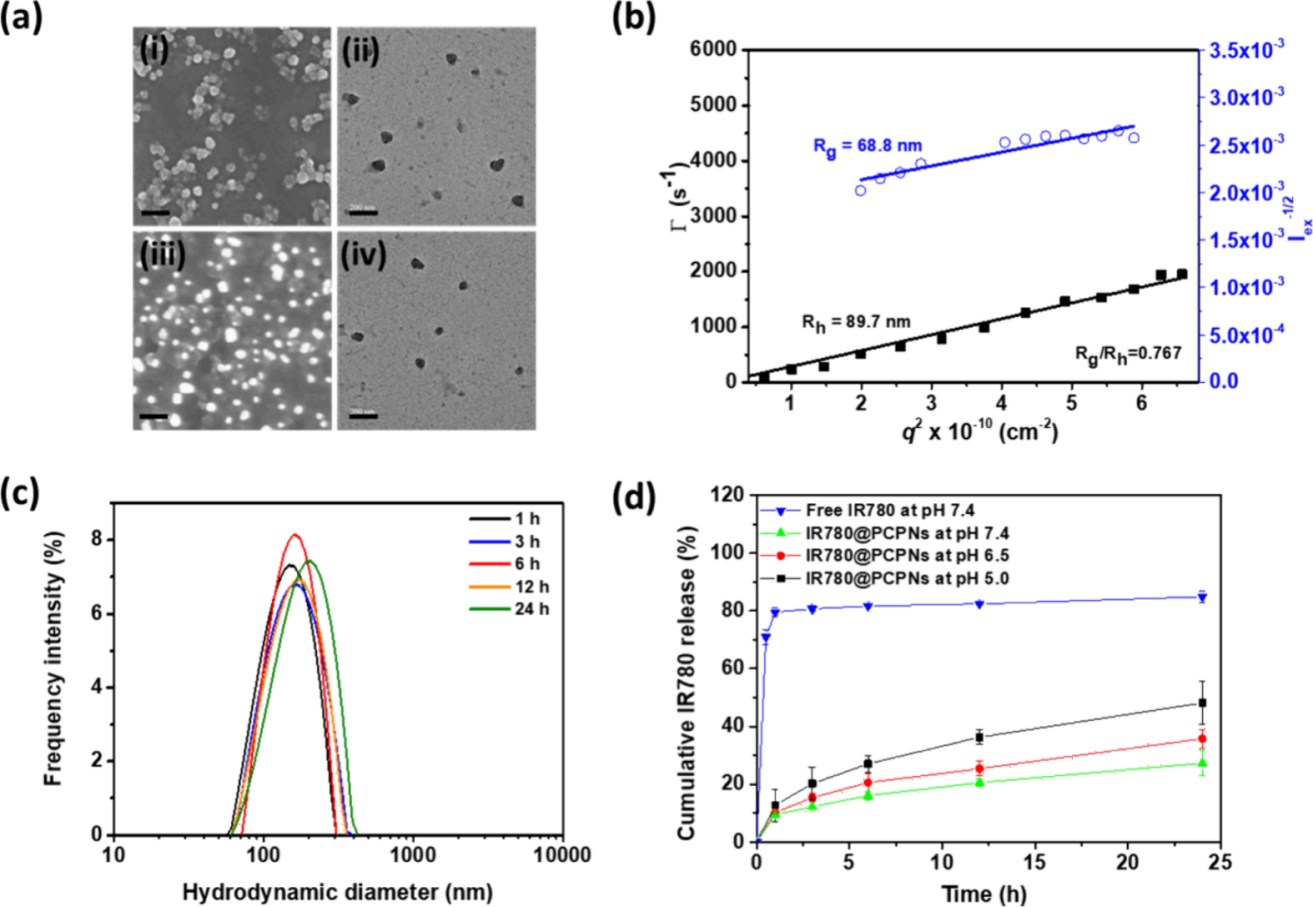
(a) SEM images of (i) PCPNs and (iii) IR780@PCPNs. TEM images of (ii) PCPNs and (iv) IR780@PCPNs. Scale bars: 200 nm. (b) Angle-dependent DLS (black square) and SLS (blue circle) measurements performed on IR780@PCPNs in pH 7.4 PBS. (c) Particle size distribution profiles of IR780@PCPNs dispersed in 10% FBS-containing pH 7.4 PBS at various intervals. (d) Cumulative IR780 release performance of IR780@PCPNs in aqueous pH 7.4, 6.5, and 5.0 solutions at 37 °C.

SEM and TEM images showed that PCPNs exhibited a well-dispersed spherical form (Figure 3a). Note that the particle sizes of the PCPNs observed by SEM or TEM were appreciably smaller than those determined by DLS due to their structural transition from the dehydration (SEM or TEM) to hydration (DLS) status. Also, a strong linear relationship between the relaxation frequency (Γ) and the square of the scattering vector (*q*^2^) observed in the angle-dependent DLS data of PCPNs in pH 7.4 PBS (Figure 3b) indicates their spherical form in aqueous solution.^40^ Also, based on the *R*_g_ of PCPNs determined by SLS to be 61 nm (Figure S5), their *R*_g_/*R*_h_ value was estimated to be ca. 0.79. Some previous studies showed that the solid sphere-like nanoparticles from the self-assembly of amphiphilic copolymers possessed an *R*_g_/*R*_h_ value of 0.78.^40,41^ Based on DLS/SLS data and TEM/SEM images, the PCPNs exhibited a solid sphere-like shape consisting of a PDA core covered by PEGylated chitosan (Scheme 1a). Furthermore, it was observed that the PCPNs dispersed in pH 7.4 PBS with or without 10% FBS at 37 °C maintained nearly unchanged particle size over 24 h (Figure S6). These findings illustrate that the hydrophilic PEGylated chitosan-rich surfaces of PCPNs could promote their colloidal stability by preventing interparticle aggregation and nonspecific protein adsorption.

Through the addition of IR780 molecules to the PCPN solution, IR780@PCPNs were attained. As revealed in the UV/vis spectra (Figure 2a), distinct from the PCPNs, the IR780@PCPNs showed the feature IR780 absorption. Moreover, the characteristic IR780 absorption of IR780@PCPNs remarkably shifted from 779 to 786 nm, being ascribed to the incorporation of IR780 molecules with PCPNs via the hydrophobic, π-π stacking, and charge interactions between cationic IR780 and negatively charged PDA. Some studies also reported a remarkable red shift in the IR780 absorption of IR780-carrying PDA-rich nanocarriers.^18,42^ Notably, the IR780 loading efficiency and content of IR780@PCPNs were determined to be ca. 96.7% and 8.8 wt % (Table 1). The mean hydrodynamic diameter of IR780@PCPNs attained at ca. 166.9 nm is somewhat larger than that of the drug-free PCPNs (Table 1 and Figure 2b).

Importantly, the IR780@PCPNs at pH 5.0 showed marked zeta potential conversion from +5 to +15 mV with prolonged time from 5 to 60 min (Figure 2f). Such an acidity-elicited zeta potential variation of IR780@PCPNs could be attributed to the following reasons. First, when the solution pH was adjusted from 7.4 to 5.0, within 5 min, the zeta potential of IR780@PCPNs was quickly converted from −6.1 to +5 mV. Obviously, in addition to the reduced dissociation of phenolic hydroxyl residues of the PDA core, as evidenced by the decrease in surface negative charges (Figure 2e), the primary amine groups of chitosan segments rapidly protonated in an acidic environment, thus leading to the increase in positive charges on the nanoparticle surfaces. Subsequently, through the acid-induced disintegration of benzoic imine of PEGylated chitosan, the PEG segments gradually detached from the surfaces of IR780@PCPNs over time, leading to increased exposure of positively charged chitosan. By contrast, the IR780@PCPNs at pH 7.4 maintained a nearly unchanged zeta potential (ca. −6.1 mV) at the same intervals due to a bit of protonation of the chitosan segments and PEG covering-induced charge shielding. Furthermore, no significant size variation of IR780@PCPNs was attained in response to a pH change from 7.4 to 5.0 over 48 h (Figure S7). This indicates that the IR780@PCPNs could retain stable colloidal stability during acidity-triggered PEG detachment.

According to TEM and SEM images (Figure 3a) and DLS/SLS data (Figure 3b), the IR780@PCPNs have a well-dispersed spherical solid-like conformation similar to PCPNs. By the hydrophilic PEGylated chitosan surfaces, the IR780@PCPNs maintained robust colloidal stability in pH 7.4 PBS with or without 10% FBS for 24 h (Figures 3c and S8). Notably, the IR780@PCPNs dispersed in pH 7.4 PBS remarkably declined IR780 premature leakage (below 20% within 6 h) compared to free IR780 molecules that quickly diffused across the dialysis tube under the same condition (over 80% within 6 h) (Figure 3d). For IR780@PCPNs at pH 7.4, the extensive hydrophobic, π–π stacking, and charge interactions between cationic IR780 and negatively charged PDA could sufficiently hinder IR780 liberation. When the solution pH was adjusted from 7.4 to 5.0, the level of cumulative IR780 liberation from IR780@PCPNs was appreciably increased. This indicates that the diminished interactions of PCPNs with IR780 molecules driven by acidity-induced protonation of chitosan segments and phenolic hydroxyl groups from PDA nanoparticles and dePEGylation could remarkably accelerate the IR780 outflow from IR780@PCPNs. Such an acidity-activated IR780 release was expected to enhance the NIR-triggered intracellular ^1^O_2_ production by increasing the contact of IR780 with endogenous oxygen.

### 3.3 Photothermal Performance and Stability of IR780@PCPNs

**Figure 4:**
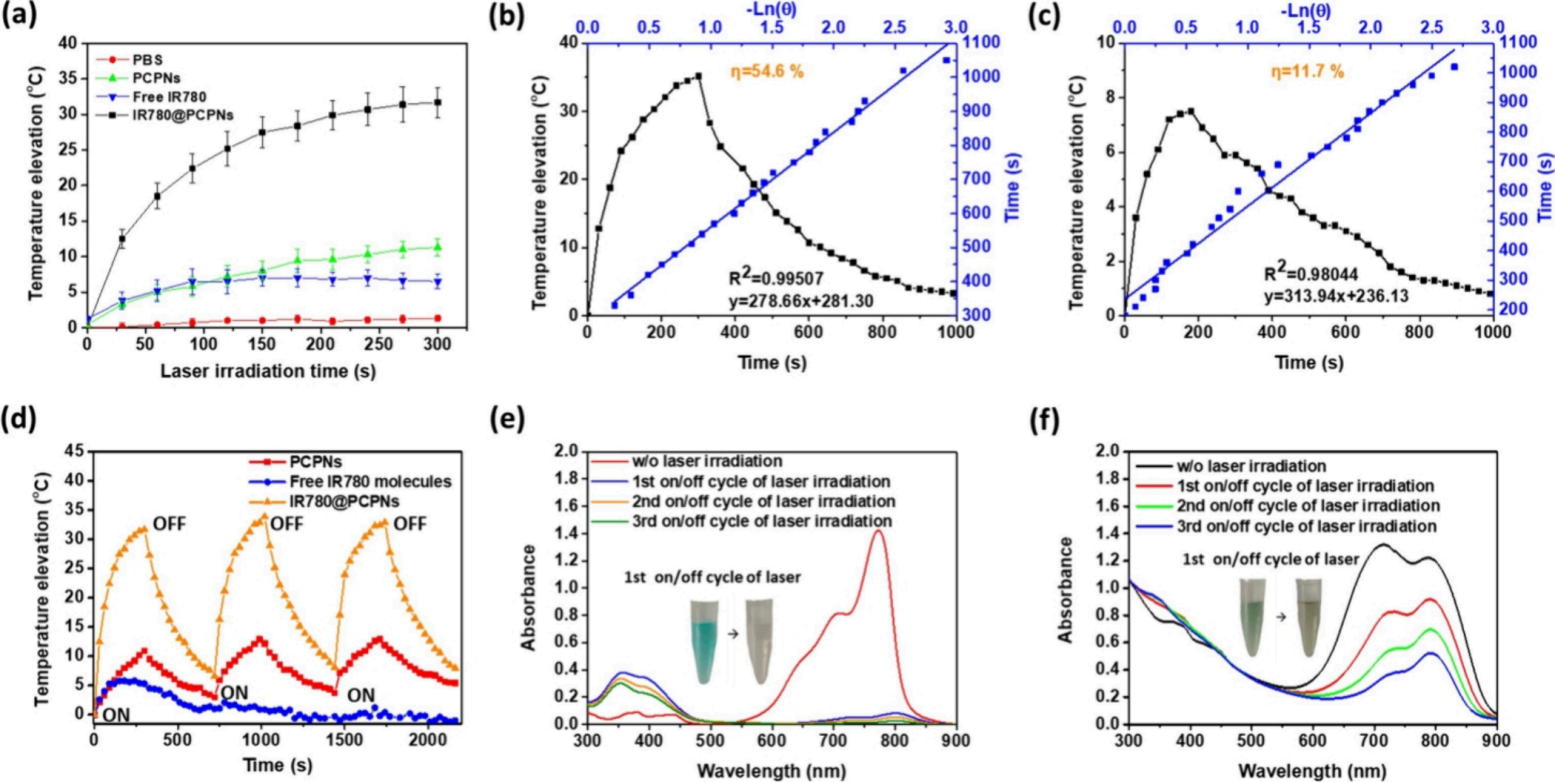
(a) Heating curves of IR780 molecules, PCPNs, and IR780@PCPNs in pH 7.4 PBS exposed to NIR laser irradiation. Photothermal performance and plot fitting of cooling time versus the negative natural logarithm of the driving force temperature during the cooling process of (b) IR780@PCPN solution and (c) free IR780 molecules (IR780 concentration: 22.2 μM). (d) Photothermal performance test for aqueous solutions of IR780 molecules, PCPNs, or IR780@PCPNs receiving three laser on/off cycles (808 nm, 1.0 W/cm^2^). UV/vis spectra of (e) the IR780 solution and (f) the IR780@PCPN solution receiving repeated laser irradiation. Inset: Photos of IR780 solution and IR780@PCPN solution before and after the first laser on/off cycle (808 nm, 1.0 W/cm^2^).

As presented in Figure 4a, with NIR laser irradiation (1.0 W/cm^2^ for 300 s), the temperature elevation of the IR780@PCPN solution was much higher than that of aqueous solutions containing either free IR780 molecules or PCPNs at the same IR780 and PCPN concentrations. According to the photothermal heating–cooling curve (Figure 4b,c), the photothermal conversion efficiency (η) of IR780@PCPNs was calculated to be 54.6%, remarkably higher compared to that of IR780 molecules (11.7%) and PCPNs (44.6%) (Figure S9). Through the inherent PDA-based photothermal property of PCPNs and the remarkable red-shifted absorption of the encapsulated IR780 molecules, the IR780@PCPNs displayed an enhanced NIR absorption capability and photothermal conversion efficiency. It is worth highlighting that IR780@PCPNs showed a photothermal conversion efficiency superior to other IR780-loaded nanoparticles reported in previous studies.^43−45^ Moreover, during NIR laser radiation, the elevation of the solution temperature was considerably amplified with an increased IR780@PCPN concentration from 85 to 340 μg/mL or enhanced laser power density from 0.5 to 1.25 W/cm^2^ (Figure S10). Notably, following three cycles of NIR laser irradiation (on/off), compared to free IR780 molecules with significantly reduced photothermal ability, the IR780@PCPNs still maintained a nearly intact photothermal effect (Figure 4d). On the other hand, after repeated NIR laser irradiation, compared to the dramatic decline in the absorption (600–800 nm) of free IR780 molecules (Figure 4e), the absorption of IR780@PCPNs was moderately decreased, indicating the reduced photoinduced degradation degree of IR780 encapsulated within PCPNs (Figure 4f). Interestingly, the IR780@PCPNs exhibited somewhat reduced IR780 absorbance post-repeated NIR irradiation but nearly unchanged photothermal effect, although this mechanism is currently unclear. Furthermore, the IR780@PCPNs remained virtually unchanged in the particle size before and after laser irradiation (Figure S11). These findings signify that the IR780@PCPNs have a prominent photothermal effect and stability, thus showing promising application in the melanoma treatment of IR780-based PTT and PDT.

### 3.4 ^1^O_2_ Production and GSH Exhaustion of IR780@PCPNs

**Figure 5:**
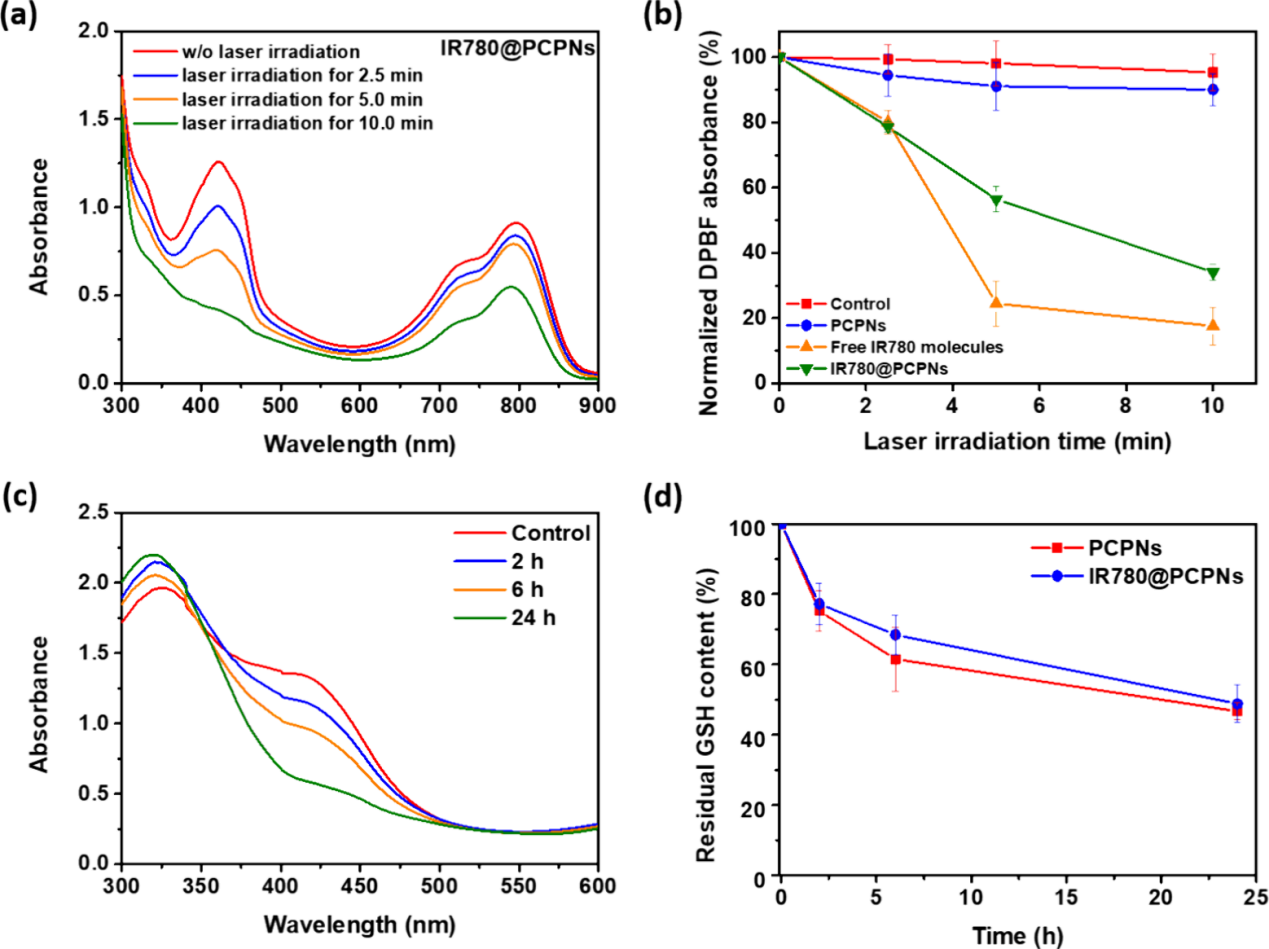
(a) UV/vis absorption spectra of DPBF in IR780@PCPNs-containing aqueous solution receiving 808 nm laser irradiation at different times. (b) Normalized absorbance of DPBF in aqueous solutions of IR780, PCPNs, and IR780@PCPNs, respectively, exposed to 808 nm laser irradiation at different times. (c) UV/vis absorption spectra of DTNB in a GSH solution treated with IR780@PCPNs for different time intervals. (d) Residual content of GSH treated with PCPNs and IR780@PCPNs for various time intervals.

The ability of IR780@PCPNs to generate ^1^O_2_ upon NIR-triggering was evaluated by using DPBF, a probe for ^1^O_2_. After reacting with ^1^O_2_, DPBF displayed a decline in absorption at around 410 nm. With 808 nm NIR laser irradiation (1.0 W/cm^2^), no significant change in the absorbance of DPBF molecules in the PCPN solution was attained (Figure S12a). In contrast, with irradiation time being prolonged from 2.5 to 10 min, the absorbance of DPBF molecules in aqueous solutions of IR780@PCPNs or IR780 molecules (IR780 concentration = 66 μM) was considerably reduced (Figures 5a and S12b). The results suggest that the IR780@PCPNs and free IR780 molecules can convert oxygen to ^1^O_2_ through the NIR-triggered IR780-based photodynamic effect. As presented in Figure 5b, with laser irradiation of 5 and 10 min, the DPBF in IR780 solution showed appreciably lower normalized absorbance than DPBF in IR780@PCPN solution, indicating that free IR780 molecules produced more ^1^O_2_ due to the effective contact of free IR780 molecules with surrounding oxygen to perform photodynamic activation. Similar results regarding the increased ^1^O_2_ production of free photosensitizers were reported elsewhere.^46,47^

Although ^1^O_2_-mediated PDT shows great potential in clinical cancer treatment, the intracellular high GSH level leads to significant scavenging of ^1^O_2_ to diminish oxidative damage, thereby lowering the cytotoxicity of PDT. To address this issue, combining the GSH-depleting functionalized nanoparticles with PDT reagents is essential. Since PDA can eliminate GSH through Michael addition,^48,49^ the GSH depletion capability of IR780@PCPNs at 37 °C was evaluated by DTNB assay. Through the thiol-exchange reaction between DTNB and GSH, 5-thio-2-nitrobenzoic acid (TNB) was produced. As shown in Figures 5c and S13, in the GSH aqueous solution pretreated with IR780@PCPNs or PCPNs, the TNB absorbance at 412 nm gradually declined as the pretreatment time was prolonged. This verifies that the PDA of PCPNs and IR780@PCPNs could scavenge GSH by covalent coupling of quinone moieties from PDA with thiol groups of GSH, thus reducing the amount of GSH reacted with DTNB. Notably, after 24 h of pretreatment, the IR780@PCPN and PCPN groups exhibited comparable residual GSH content (Figure 5d), suggesting that the encapsulation of IR780 molecules into PCPNs could not impact the GSH consumption performance of PCPNs.

### 3.5 Cellular Uptake and Intracellular ^1^O_2_ Production

**Figure 6:**
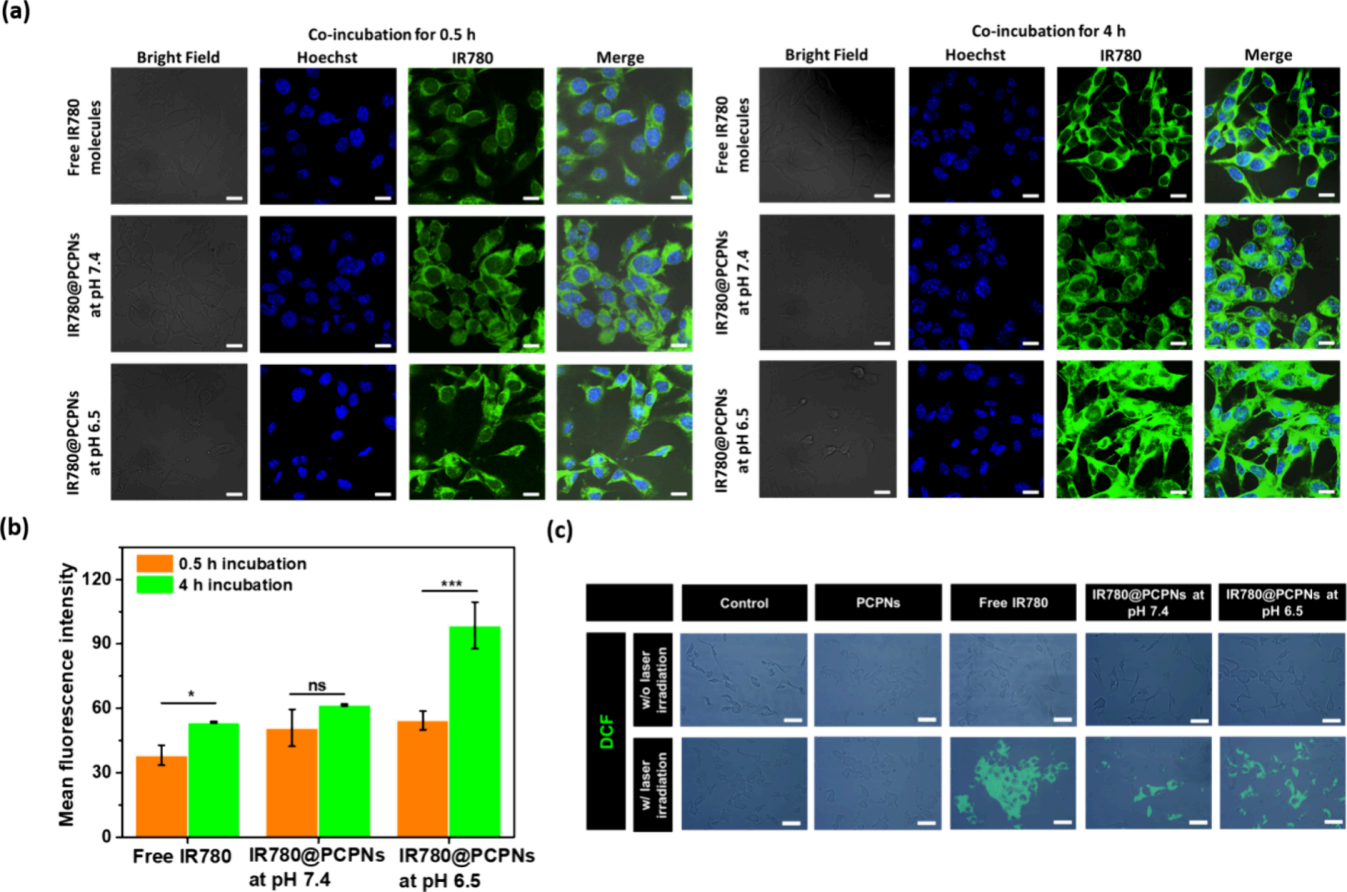
(a) CLSM images and (b) intracellular mean IR780 fluorescence intensity of B16F10 cells incubated with free IR780 molecules at pH 7.4 or IR780@PCPNs at pH 7.4 or 6.5 for 0.5 and 4 h, respectively, at 37 °C (IR780 = 2.5 μM). Scale bars: 15 μm. (c) DCF fluorescence images of B16F10 cells receiving different treatments. Scale bars: 50 μm.

In order to investigate the effect of acidity-triggered dePEGylation on the cellular uptake of IR780@PCPNs, their internalization by B16F10 cells was observed by CLSM. Free IR780 molecules were employed for comparison. As shown in Figure 6a,b, when the coculture time was prolonged from 0.5 to 4 h, the intracellular IR780 fluorescence signals of B16F10 cells treated with IR780@PCPNs at pH 6.5 mimicking a weakly acidic tumor microenvironment were appreciably enhanced compared to those of cells receiving IR780@PCPNs at pH 7.4. This signifies that the dePEGylation and positive charge exposure of IR780@PCPNs upon acidity-activated hydrolysis of the benzoic imine bond and chitosan protonation could enhance their affinity for B16F10 cells, thus increasing their cellular uptake. Similar results regarding the boosted cellular internalization of nanoparticles by stimuli-triggered dePEGylation were also reported elsewhere.^31−36^ By contrast, in pH 7.4 culture medium, the outer PEG segments of IR780@PCPNs markedly retarded their cellular internalization. Furthermore, with 4 h incubation, B16F10 cells incubated with free IR780 molecules showed remarkably lower IR780 fluorescence signals compared to those of cells exposed to IR780@PCPNs at pH 6.5, being ascribed to the immense aggregation of hydrophobic IR780 during the coculture process to hinder the cellular internalization.

Next, the DCFH-DA probe, a ROS indicator,^50^ was utilized to assess the intracellular ^1^O_2_ production of B16F10 cells treated with free IR780 molecules and IR780@PCPNs, respectively. In the absence of NIR irradiation, B16F10 cells incubated with free IR780 molecules, PCPNs, or IR780@PCPNs showed few DCF fluorescences (Figure 6c), indicating the lack of intracellular ^1^O_2_. With irradiation of the NIR laser, B16F10 cells receiving IR780 molecules (IR780 concentration = 2.5 μM) showed remarkably enhanced DCF fluorescence, illustrating the conversion of endogenous oxygen to ^1^O_2_ via IR780-based photodynamic activity. Importantly, exposed to NIR laser irradiation, B16F10 cells cocultured with IR780@PCPNs at pH 6.5 displayed stronger DCF fluorescence signals than cells receiving the counterpart at pH 7.4. Undoubtedly, the acid-triggered PEG detachment of IR780@PCPNs can augment their uptake by B16F10 cells, thereby efficiently increasing intracellular IR780 transport and generating intracellular ^1^O_2_ by the IR780-mediated photodynamic effect. In contrast, the lowered internalization of IR780@PCPNs by B16F10 cells at pH 7.4 due to PEG interfering decreased intracellular IR780 delivery, thereby reducing NIR-triggered ^1^O_2_ production. Furthermore, after laser irradiation, compared with the B16F10 cells receiving IR780@PCPNs at pH 6.5, the IR780-treated B16F10 cells showed somewhat higher DCF fluorescence intensity. This could be attributed to that free IR780 molecules sufficiently converted intracellular oxygen to ^1^O_2_. In contrast, the steric barriers of IR780@PCPNs partly limited the contact of IR780 molecules with oxygen, thereby decreasing the ^1^O_2_ production.

### 3.6 Mitochondria Damage and In Vitro Anticancer Potency

**Figure 7:**
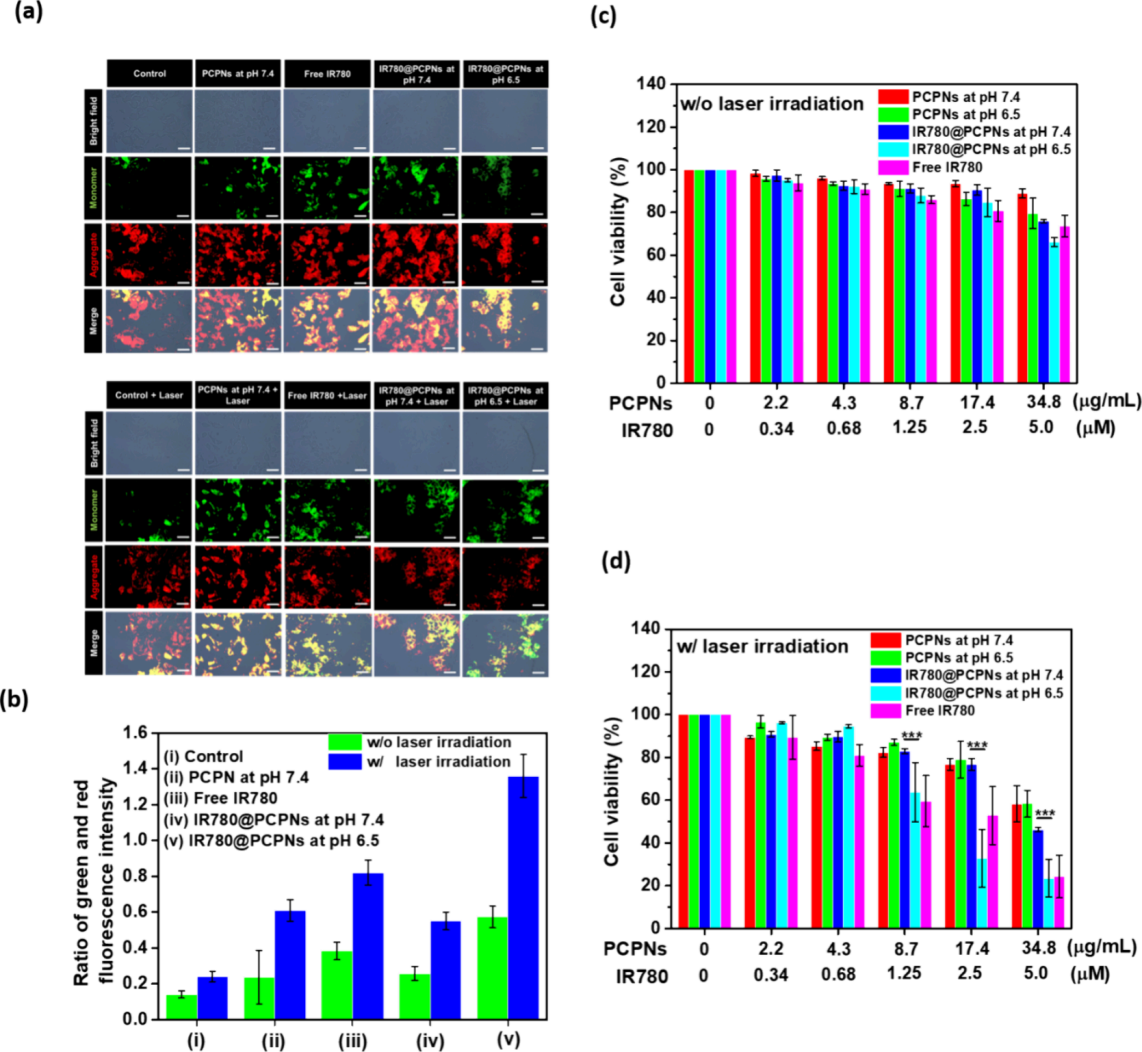
(a) JC-1 staining images and (b) ratio of green and red fluorescence intensities of B16F10 cells subjected to different treatments. Scale bars: 50 μm. Viability of B16F10 cells treated with PCPNs, IR780@PCPNs at pH 7.4 or 6.5, or free IR780 molecules at pH 7.4 without laser irradiation (c) or laser irradiation (d).

The mitochondrial damage has been reported to be a vital and characteristic mark of ROS-involved apoptosis.^51,52^ To investigate the effects of ^1^O_2_ generated from IR780@PCPNs on the mitochondria of B16F10 cells, JC-1 was employed to indicate mitochondrial membrane potential. JC-1 forms an aggregate (in healthy mitochondria) with red fluorescence. As membrane potential declines due to apoptosis, JC-1 becomes monomers, showing green fluorescence.^51−53^ Without laser irradiation, B16F10 cells cocultured with IR780 molecules, PCPNs, or IR780@PCPNs exhibited strong red fluorescence and a low ratio of green and red fluorescence intensity (Figure 7a,b), indicating intact mitochondrial function. Interestingly, with laser irradiation, a somewhat decreased red fluorescence intensity and increased ratio of green and red fluorescence intensity were observed in the PCPN-treated B16F10 cells, indicating mitochondria destruction by NIR-triggered PCPN-based hyperthermia. Such a hyperthermia-elicited functional disorder of the mitochondria in different cancer cell lines was reported in other studies.^54,55^ Notably, with NIR laser irradiation, B16F10 cells receiving IR780@PCPNs at pH 6.5 exhibited significantly reduced red fluorescence signals and a prominent amplified ratio of green and red fluorescence intensity compared to cells incubated with IR780@PCPNs or PCPNs at pH 7.4. This suggests that the promoted internalized IR780@PCPNs upon acidity-elicited dePEGylation could remarkably damage mitochondria by NIR-triggered ^1^O_2_ generation and hyperthermia. Moreover, considering the short diffusion distance and half-life of ^1^O_2_, the mitochondria-targeting capability of free IR780^56,57^ and its better ^1^O_2_-producing ability (Figure 5b), the acidity-activated IR780 liberation of IR780@PCPNs was assumed to promote the targeting of IR780 to mitochondria and augment the ^1^O_2_ generation and mitochondria damage (Scheme 1b).

To investigate the effect of acidity-triggered dePEGylation of IR780@PCPNs on their PDT/PTT-mediated anticancer capability, the viability of B16F10 cells incubated with IR780@PCPNs at pH 7.4 or 6.5 and exposed to laser irradiation was examined using the MTT test. As presented in Figure 7c, without laser irradiation, B16F10 cells incubated with PCPNs (2.2∼17.4 μg/mL), free IR780, or IR780@PCPNs (IR780 concentration of 0.34∼2.5 μM) showed quite high cell viability (over 80%). As the IR780 concentration was increased to 5.0 μM, the viability of B16F10 cells treated with IR780@PCPNs or free IR780 somewhat declined to below 80%, indicating a little cytotoxicity of IR780 on cancer cells. In contrast, under laser irradiation, the viability of B16F10 cells incubated with free IR780 molecules or IR780@PCPNs appreciably reduced with increased IR780 concentration, while the viability of cells treated with PCPNs slightly decreased as the concentration of PCPNs was increased to 34.8 μg/mL (Figure 7d). Based on these findings, compared to single PTT delivered by PCPNs, the combined PTT and PDT based on free IR780 molecules or IR780@PCPNs displayed a prominent anticancer effect on B16F10 cells. Notably, with the culture milieu pH being changed from 7.4 to 6.5, the viability of IR780@PCPN-treated B16F10 cells with NIR laser irradiation was further reduced. Furthermore, based on the cytotoxicity data (Figure 7d), the IR780 concentration needed to achieve a 50% inhibition of cell growth (IC_50_ value) for IR780@PCPNs at pH 6.5, estimated to be 1.32 μM, is appreciably 3.6 times lower compared to that (4.69 μM) of the counterparts at pH 7.4. This suggests that the acidity-triggered PEG detachment of IR780@PCPNs could further promote their cellular uptake to increase intracellular IR780 delivery, thus eliciting cell death upon NIR-activated ^1^O_2_ production and hyperthermia. It should be highlighted that the IC_50_ of IR780@PCPNs at pH 6.5 is somewhat lower than that (2.74 μM) of free IR780, illustrating the anticancer potency of IR780@PCPNs superior to free IR780 due to the promoted intracellular IR780 delivery, photothermal effect, and stability of IR780 molecules. On the other hand, without NIR laser irradiation, the healthy WS1 cells incubated with free IR780, PCPNs, and IR780@PCPNs, respectively, showed high viability (over 90%) (Figure S14). This signifies that the IR780@PCPNs have little cytotoxicity on the normal cells without laser irradiation. According to the above results, the NIR-triggered PTT and PDT delivered by IR780@PCPNs are expected to selectively kill cancer cells and reduce the adverse effect on normal cells.

## 4 Conclusions

To realize effective melanoma treatment by amplifying the anticancer effect of the combined PTT and PDT, in this work, the acidity-triggered PEG detachable PCPNs composed of a PDA core surrounded by PEGylated chitosan were developed as IR780 carriers. The IR780@PCPNs exhibited a mean hydrodynamic diameter of ca. 166.9 nm and a solid-like spherical shape. Through the acidity-activated cleavage of benzoic imine, the PEG segments were detached from the IR780@PCPNs, exposing a positively charged surface. Furthermore, the IR780@PCPNs showed accelerated IR780 release in response to a pH reduction from 7.4 to 5.0. Compared to free IR780 molecules, the IR780@PCPNs displayed superior photothermal conversion efficiency, colloidal stability, and photothermal stability. Also, IR780@PCPNs mediate the depletion of GSH by the Michael addition between PDA and GSH and produce ^1^O_2_ under NIR laser irradiation. Notably, under a culture condition of pH 6.5, imitating an acidic tumor microenvironment, the internalization of IR780@PCPNs by B16F10 melanoma cells was efficiently promoted via acidity-triggered dePEGylation. With NIR laser irradiation, the endocytosed IR780@PCPNs generated hyperthermia and sufficient ^1^O_2_ to disrupt mitochondria, thus killing cancer cells. In conclusion, the developed IR780@PCPNs showed promoted cellular uptake by acidity-triggered dePEGylation, thus amplifying the anticancer efficacy of combined PDT and PTT on melanoma cells. This may provide a meaningful strategy for future clinical melanoma treatment.

## Supporting Information Available

Synthetic pathway of benzoic imine-containing PEGylated chitosan adducts, photos of the aqueous solutions containing bare PDA particles and PCPNs, respectively, N 1s XPS spectrum of PDA, TGA profiles of PEGylated chitosan adducts, PDA particles, and PCPNs, Berry plot for *R*_g_ and angle-dependent correlation function of *R*_h_ of PCPNs in pH 7.4 PBS, particle size distribution profiles of PCPNs dispersed in pH 7.4 PBS or 10% FBS-containing pH 7.4 PBS for various time intervals, particle size distribution profiles of IR780@PCPNs dispersed in pH 7.4 PBS and pH 5.0 acetate buffer for 48 h, particle size distribution profiles of IR780@PCPNs dispersed in pH 7.4 PBS for different time intervals, temperature change of PCPN solution for single laser on/off cycle, temperature elevation profiles of IR780@PCPNs with different concentrations in PBS exposed to laser irradiation and IR780@PCPNs in pH 7.4 PBS with NIR laser irradiation of different laser power densities, particle size distribution profiles of IR780@PCPNs dispersed in pH 7.4 PBS with or without NIR laser irradiation, UV/vis spectra of DPBF in PCPN solution or IR780 solution receiving laser irradiation of different irradiation times, UV/vis spectra of DTNB in GSH solution treated with PCPNs at 37 °C for various time intervals, and viability of WS1 cells exposed to various formulations without laser irradiation (PDF)
