# Supplementary material for: Enhanced Intracellular IR780 Delivery by Acidity-Triggered PEG-Detachable Hybrid Nanoparticles to Augment Photodynamic and Photothermal Combination Therapy for Melanoma Treatment
Source: ACS Appl Bio Mater. 2025 Apr 12;8(5):3995–4007. doi: 10.1021/acsabm.5c00144 (PMC12093379; doi:10.1021/acsabm.5c00144)
Supplement: Supplementary file 1 — mt5c00144_si_001.pdf [file mt5c00144_si_001.pdf]

Supporting Information

Enhanced Intracellular IR780 Delivery by Acidity-Triggered PEG-Detachable Hybrid Nanoparticles to Augment Photodynamic and Photothermal Combination Therapy for Melanoma Treatment

Min-Chen Tsai [a], Lun-Yuan Hsiao [a], Yen-Hsuan Chang [a], Yu-Hsin Chen [a], Shang-Hsiu Hu [b], Chun-Yu Hung [c], Wen-Hsuan Chiang [a,d]*

[a] Department of Chemical Engineering, National Chung Hsing University, Taichung 402, Taiwan.

[b] Department of Biomedical Engineering and Environmental Sciences, National Tsing Hua University, Hsinchu 300, Taiwan.

[c] Department of Orthopedic Surgery, Jen-Ai Hospital, Taichung 402, Taiwan.

[d] i-Center for Advanced Science and Technology (iCAST), National Chung Hsing University, Taichung 402, Taiwan.

* Corresponding authors: whchiang@dragon.nchu.edu.tw (W.-H. Chiang)

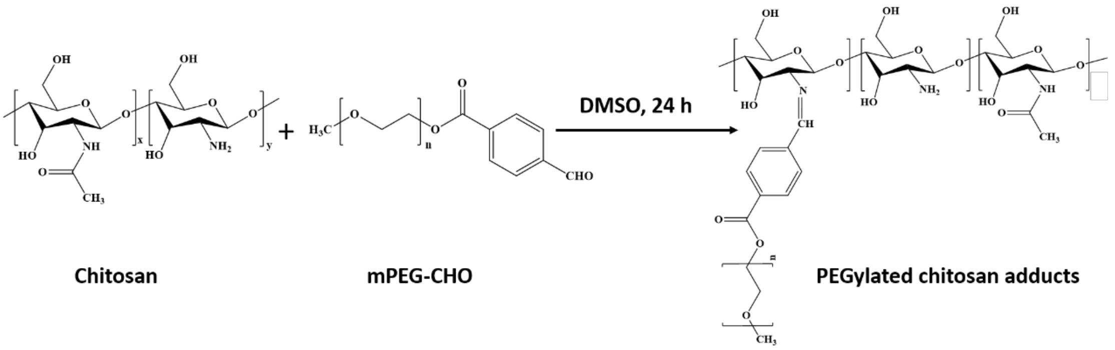

Figure S1. Synthetic pathway of benzoic imine-containing PEGylated chitosan adducts.

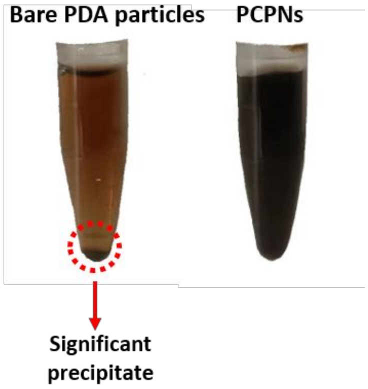

Figure S2. Photos of the aqueous solutions containing bare PDA particles and PCPNs, respectively.

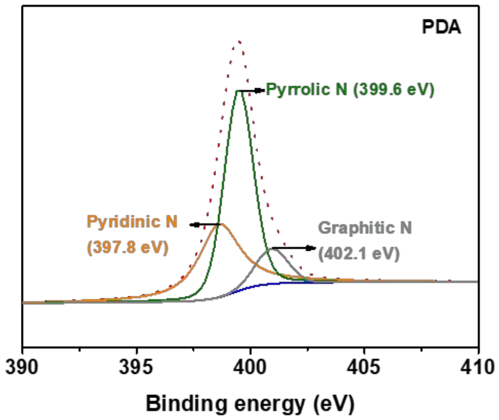

Figure S3. N1s XPS spectrum of PDA.

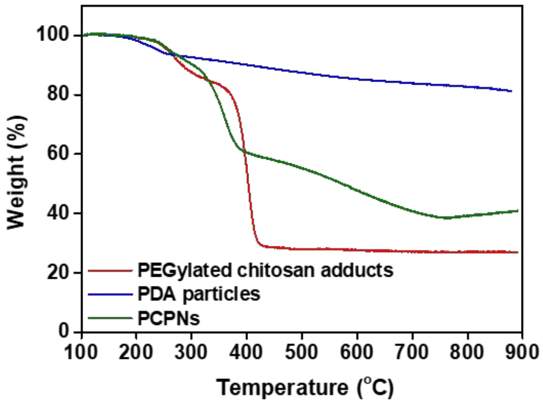

Figure S4. TGA profiles of PEGylated chitosan adducts, PDA particles, and PCPNs.

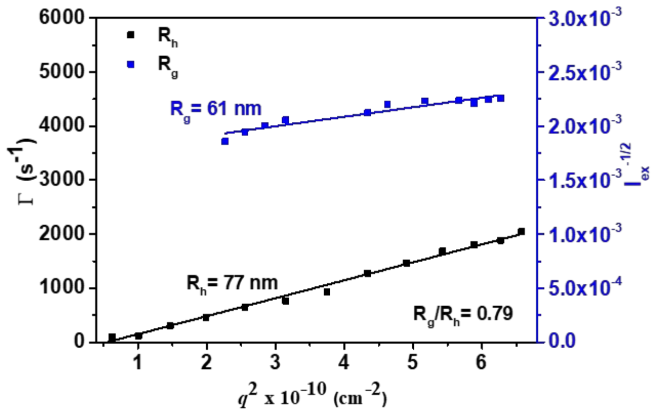

Figure S5. Berry plot for R_g and angle-dependent correlation function of R_h of PCPNs in pH 7.4 PBS.

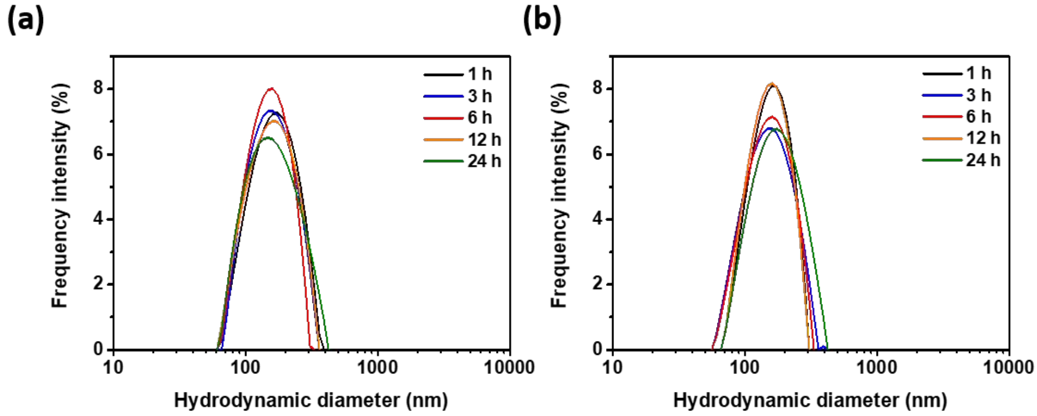

Figure S6. Particle size distribution profiles of PCPNs dispersed in (a) pH 7.4 PBS and (b) 10 % FBS-containing pH 7.4 PBS at various time intervals.

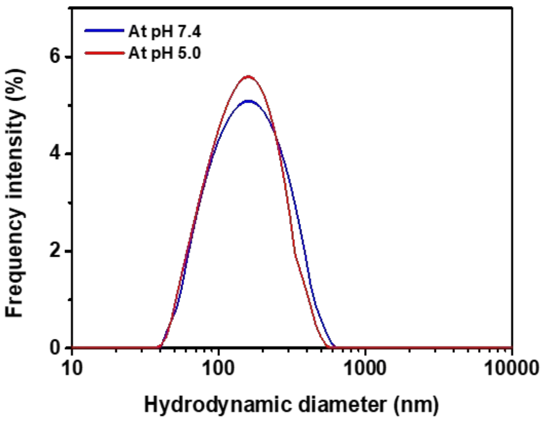

Figure S7. Particle size distribution profiles of IR780@PCPNs dispersed in pH 7.4 PBS and pH 5.0 acetate buffer for 48 h.

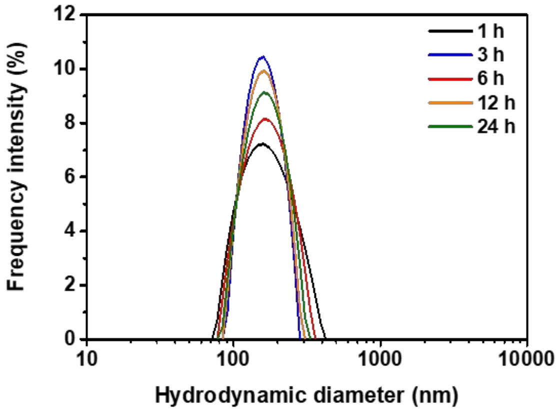

Figure S8. Particle size distribution profiles of IR780@PCPNs dispersed in pH 7.4 PBS at different time intervals.

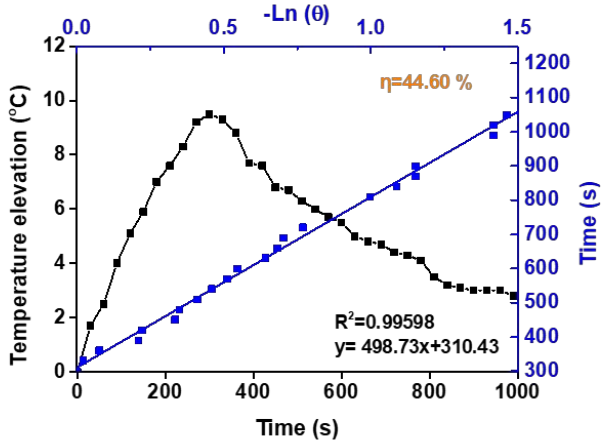

Figure S9. Temperature elevation profile of PCPN solution (PCPN concentration: 155 μg/mL) for single on/off cycle of 808 nm laser irradiation (1.0 W/cm^2), and plot of cooling time versus the negative logarithm of the temperature driving force.

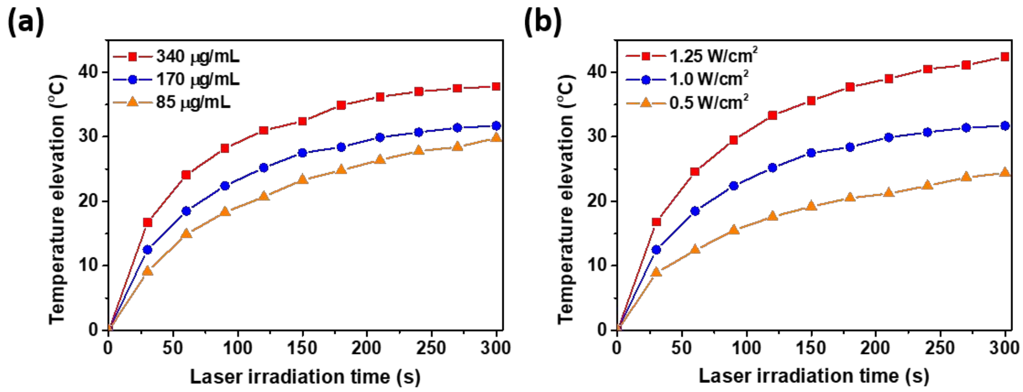

Figure S10. Temperature elevation profiles of (a) IR780@PCPNs with different concentrations in pH 7.4 PBS exposed to 808 nm NIR laser irradiation (1.0 W/cm^2) and (b) IR780@PCPNs (170 μg/mL) in pH 7.4 PBS with NIR laser irradiation of different laser power densities.

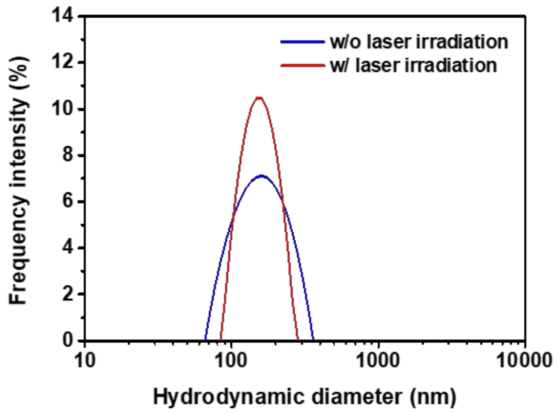

Figure S11. Particle size distribution profiles of IR780@PCPNs dispersed in pH 7.4 PBS with or without NIR laser irradiation.

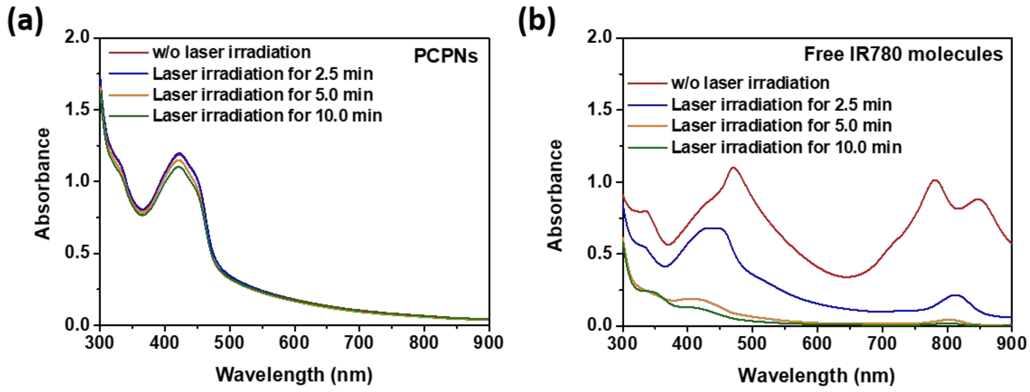

Figure S12. UV/Vis spectra of DPBF molecules in aqueous solutions containing (a) PCPNs and (b) free IR780 molecules receiving 808 nm laser irradiation of different irradiation times (1.0 W/cm^2).

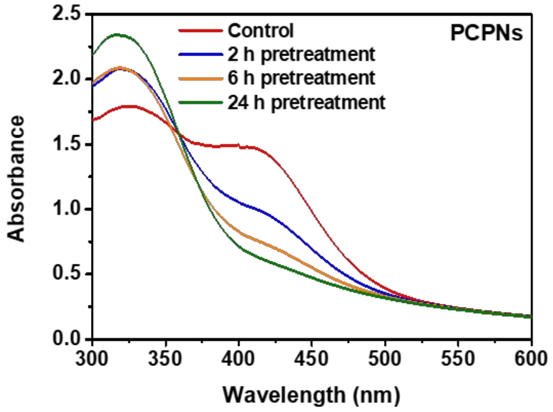

Figure S13. UV/Vis spectra of DTNB molecules in GSH solution pretreated with PCPNs at 37 ^{o}C for various time intervals.

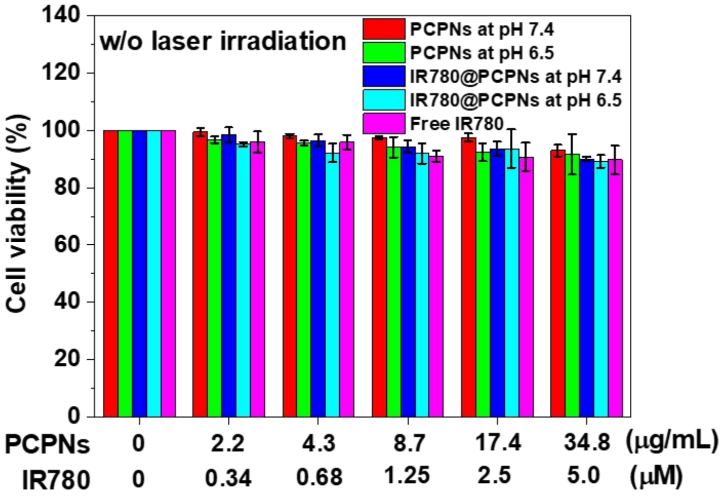

Figure S14. Viability of WS1 cells receiving different formulations without laser irradiation.
